# Detection of Lassa Virus, Mali

**DOI:** 10.3201/eid1607.100146

**Published:** 2010-07

**Authors:** David Safronetz, Job E. Lopez, Nafomon Sogoba, Sékou F. Traore’, Sandra J. Raffel, Elizabeth R. Fischer, Hideki Ebihara, Luis Branco, Robert F. Garry, Tom G. Schwan, Heinz Feldmann

**Affiliations:** Author affiliations: National Institutes of Health, Hamilton, Montana, USA (D. Safronetz, J.E. Lopez, S.J. Raffel, E.R. Fischer, H. Ebihara, T.G. Schwan, H. Feldmann);; University of Bamako, Bamako, Mali (N. Sogoba, S.F. Traore’);; Tulane School of Medicine, New Orleans, Louisiana, USA (L. Branco, R.F. Garry);; Autoimmune Technologies LLC, New Orleans (L. Branco)

**Keywords:** Viruses, arenavirus, Lassa fever, Lassa virus, rodent-borne infections, Mastomys natalensis, field studies, Mali, dispatch

## Abstract

To determine whether Lassa virus was circulating in southern Mali, we tested samples from small mammals from 3 villages, including Soromba, where in 2009 a British citizen probably contracted a lethal Lassa virus infection. We report the isolation and genetic characterization of Lassa virus from an area previously unknown for Lassa fever.

Lassa fever is an acute viral infection associated with a wide spectrum of disease manifestations, which range from mild to hemorrhagic fever characterized by multiorgan failure (*1*). The etiologic agent of Lassa fever is Lassa virus (LASV, family *Arenaviridae*, genus *Arenavirus*), which is maintained in its natural rodent reservoir, the multimammate rat (*Mastomys natalensis*) (*1*–*3*). Although *M. natalensis* rats are ubiquitous in many parts of sub-Saharan Africa, infected rodents have only been reported in West African countries, most commonly Nigeria, Sierra Leone, and Guinea (*3**–**5*). Consequently, cases of Lassa fever mainly occur in regions in which the virus is endemic, consisting of those 3 countries and Liberia, with an annual incidence ranging from 300,000 to 500,000 cases and ≈5,000 deaths (*6*). Serologic evidence of LASV infections has also been reported from other West and Central African countries (*5**,**7**–**9*). Additionally, LASV has been introduced into Europe and North America several times, making Lassa fever one of the most prominent imported exotic viral hemorrhagic fevers, which strongly affects public health systems (*9*).

In 2009, Lassa fever was diagnosed postmortem in a young man with a 10-day history of fever who had been evacuated from Mali to London (*10*). The patient had no travel history to any LASV-endemic region, which suggests that he contracted the infection in Mali, most likely while working in the village of Soromba (Figure 1). In this report, we provide evidence that LASV is circulating in *M. natalensis* in southern Mali, thereby expanding the geographic distribution of LASV in West Africa and posing a risk to humans in an area previously unknown for Lassa fever. The study was carried out in accordance with a protocol approved by an Institutional Animal Care and Use Committee of the US National Institutes of Health.

**Figure 1:**
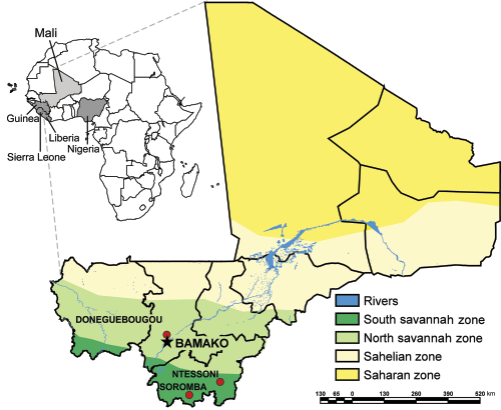
Ecozones of Mali and locations where small mammals were trapped in June 2009. Inset shows location of Mali in relation to countries where Lassa virus is endemic (shaded).

## The Study

From June 5 through 14, 2009, a total of 103 small mammals (trap success rate of 17.6%) were live trapped and sampled from 3 villages in Mali (Figure 1; Table): N’Tessoni (11°2′0′′N, 5°59′0′′W), Soromba (10°35′0′′N, 7°9′0′W), and Doneguebougou (12°48′21′′N, 7°59′0′′W). Pertinent information was recorded for each animal, and ear punch, heart, blood, lung, and liver samples were collected. *Mastomys* sp. Rats were the predominant rodent captured (82/103, 79.6%), with all but 4 of these animals identified as *M. natalensis* rat by cytochrome B sequence analysis of DNA isolated from ear punch specimens (*11*; GenBank accession nos. HM130517–HM130519).

**Table:** Results of investigation of small mammals trapped in 3 villages in Mali, 2009

Village	No. total captures (trap success, %)*	No. non–*Mastomys* spp. captures	No*. Mastomys* spp. captures (trap success, %)	No. (%) Lassa-positive *M. natalensis* mice
N’Tessoni	25 (14.9)	8†	17 (10.1)‡	0
Soromba	25 (15.0)	0	25 (15.0)	6 (24)
Doneguebougou	53 (21.1)	13†	40 (15.9)‡	0

*Trap success is defined as the total number of individual rodents captured divided by the cumulative total of traps set at each location, multiplied by 100. †Non-*Mastomys* species captured included 1 *Praomys daltoni* (Dalton’s mouse), 3 *Mus musculoides* (Temminck’s mouse), 3 *Crocidura olivieri* (giant shrew), and 1 *Arvicanthis niloticus* (grass rat) from N’Tessoni and 1 *Praomys daltoni*, 1 *Rattus rattus* rat, 1 *Arvicanthis niloticus*, 3 *Crocidura viaria* (savannah path shrew), and 7 *Crocidura olivieri* from Doneguebougou. ‡ Two *Mastomys* spp. rodents captured in N’Tessoni and Doneguebougou were identified as *M. erythroleucus* by cytochrome B sequencing.

Total RNA was extracted from tissue specimens and blood by using RNeasy or QIAamp viral RNA kits (QIAGEN, Valencia, CA, USA), respectively, and screened for the presence of LASV RNA by using a SYBR-green based, real-time reverse transcription–PCR assay that amplifies a 195-bp portion of the small genomic segment (primers Gc656s: 5′-ATTGCTCTTGACTCAGGCC-3′ and Gc851as: 5′-GTGTCCATGTGAATGTGCCTA-3′; TIB Molbiol GBH, Adelphia, NJ, USA). Six infected rodents were identified, each of which had LASV-positive lung, liver, and blood specimens. Positive rodents were genetically identified as *M. natalensis*, and all were captured from Soromba, for a village prevalence of 24% (6/25; Table). Four (66.7%) of 6 infected rodents were male and 5 (83.3%) of 6 were adult. All 6 infected animals were captured indoors.

**Figure A1:**
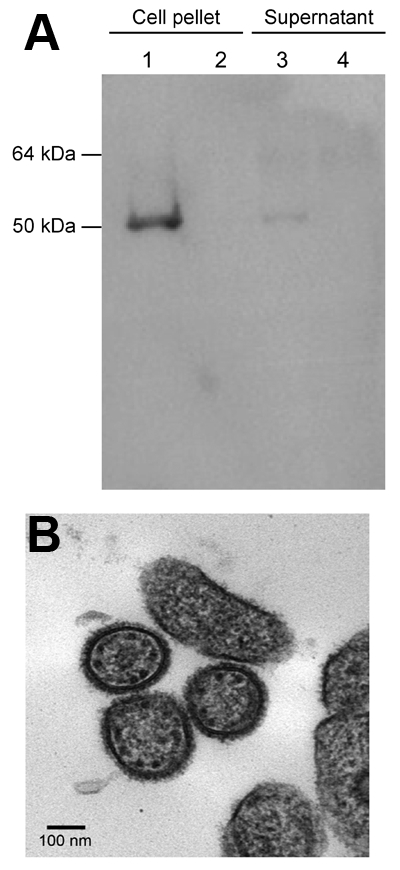
A) Confirmation of isolation of Lassa virus (LASV) Soromba-R by using immunoblot analysis of cell pellets and supernatant from LASV Soromba-R–infected Vero E6 cells (lanes 1 and 3) or uninfected controls (lanes 2 and 4) with a LASV nucleoprotein–specific monoclonal antibody. B) Electron micrograph of LASV particles.

Tissue homogenates were prepared from selected LASV-positive animals and passaged twice on subconfluent monolayers of Vero E6 cells. After 2 passages, no discernible cytopathic effect was observed, although LASV RNA was detected by reverse transcription–PCR in cells and supernatant. Virus isolation was confirmed by immunoblot analysis in infected cells and supernatant by using a LASV nucleoprotein-specific monoclonal antibody, which detected a 50–55-kDa protein consistent in size with the LASV nucleoprotein (*12**;* Figure A1, panel A, www.cdc.gov/EID/content/16/7/1123-appF.htm). Furthermore, electron microscopy performed on infected cells showed viral particles consistent in size and morphologic features with an arenavirus (Figure A1, panel B).

**Figure 2:**
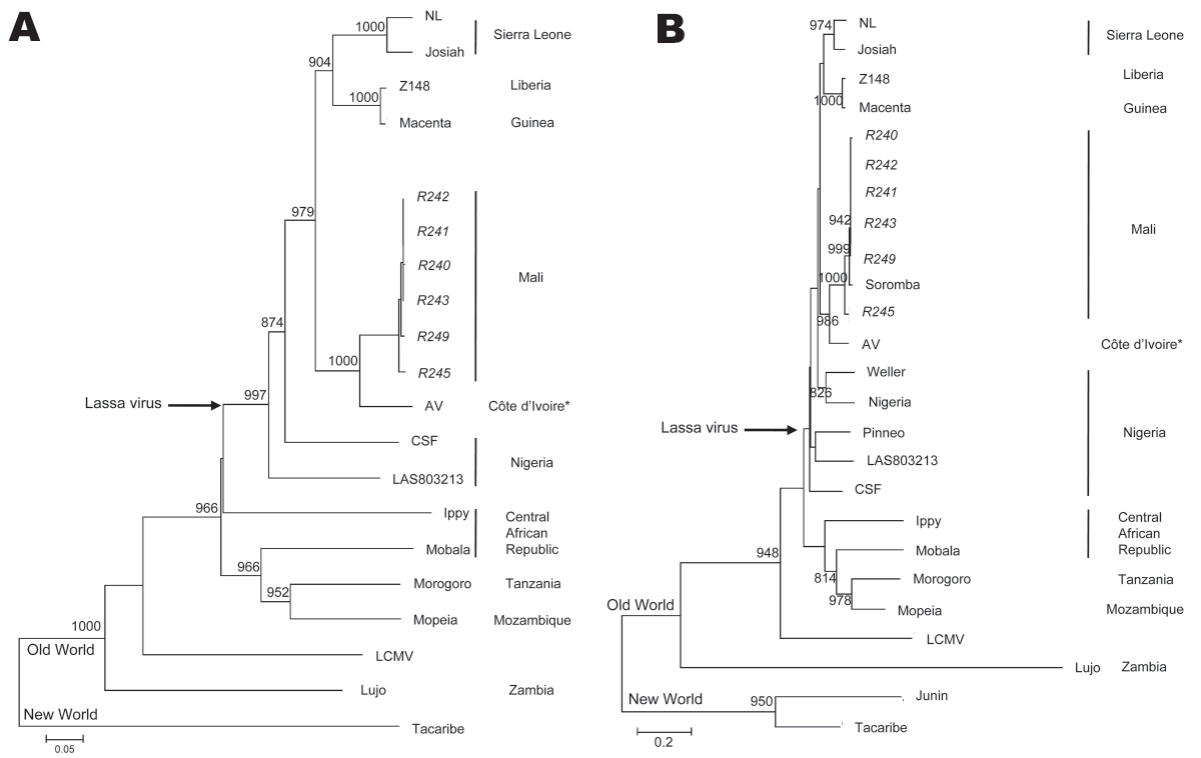
Phylogenetic analysis of Lassa virus conducted on A) a 754-bp fragment of the polymerase gene (large genomic segment nucleotide positions 3427–4180) and B) a 771-bp fragment of the glycoprotein precursor (small genomic segment nucleotide positions 2526–3296). The fragments were amplified from infected rodent tissues with sequence analysis accomplished with PHYLIP version 3.69 software (http://evolution.genetics.washington.edu/phylip.html) by using the neighbor-joining method with 1,000 replicates of bootstrap. Sequences were compared with the following arenavirus sequences (small and large segment GenBank accession nos.): Tacaribe (NC_004293, NC_004292), Lujo (NC_012776, NC_012777), Lymphocytic choriomeningitis virus (LCMV, strain Armstrong, AY847350,J04331), Ippy (NC_007905, NC_007906), Mobala (NC_007903, NC_007904), Morogoro (NC_013057, NC_013058), Mopeia (NC_006575, NC_006574), and Lassa virus strains 803213 (AF803213, AY693640), CSF (AF333969, AY179174), NL (AY179173, AY179172), Josiah (AY628203, U63094), Z148 (AY628205, AY628204), Macenta (AY628201, AY628200), and AV (AF246121, AY179171). Small segment analysis also included Lassa virus strains LP (AF18185), Weller (AY628206), Nigeria (M36544), the sequence generated from the imported case of Lassa fever from Mali (FJ824031), and Junin virus (NC_005081). Bootstrap values >800 are shown. *Whether the AV strain of Lassa virus originated from Côte d’Ivoire, Burkina Faso, or Ghana is not clear. Italicized names represent sequences generated as part of these studies. Scale bars indicate 5% (A) or 20% (B) nucleotide divergence.

An ≈800-nt fragment of the LASV polymerase gene was amplified from the isolated virus and the tissues of infected rodents by using a pan–Old World arenavirus assay (*13*). Additionally, an 873-bp fragment of the LASV glycoprotein gene, corresponding to the sequence from a British Lassa fever patient (*10*), was amplified from the same samples by using primers Gc F1 5′-GCATTTTAATTCAGCCTCAATTAAC-3′ and Gc R1 5′-ATGGGGCAGATTGTGACATTCTTTC-3′. Amplicons were sequenced (GenBank accession nos. GU573541–GU573546 and GU573547–GU573552 for polymerase and glycoprotein fragments, respectively) and aligned with previously described arenavirus sequences by using ClustalX version 2.0.10 software (www.clustal.org). Nucleotide sequences from the isolated LASV were indistinguishable from the sequences generated from the infected rodent tissues from which they were derived (data not shown). Phylogenetic analysis of the glycoprotein sequences confirmed that all rodent-derived LASV sequences belonged to the same genetic clade as the sequence of the imported Lassa fever case in the United Kingdom. Polymerase fragment sequences confirmed that this clade is most closely related to the previously described AV strain of LASV that originated from the neighboring countries of Côte d’Ivoire, Burkina Faso, or Ghana (*14*; Figure 2).

## Conclusions

This study demonstrates that *M. natalensis* rats in Mali carry a genetically unique strain of LASV (proposed name Soromba-R). Genetically, Soromba-R is nearly indistinguishable from the nucleotide sequence generated from a lethal case of Lassa fever imported from Mali to the United Kingdom, which supports the epidemiologic data that the infection occurred in the village of Soromba in southern Mali (*10*). The Soromba-R strain of LASV is genetically closely related to the AV strain, which was isolated from an imported lethal Lassa fever case in a patient who contracted the infection while traveling in neighboring countries (*14*). Thus, this study provides evidence for an expanded region of LASV endemicity in West Africa.

On the basis of Mali’s geographic proximity to countries where LASV is endemic, it should not be surprising that LASV is circulating there. Cases of Lassa fever are diagnosed annually in Guinea, and sporadic cases have been diagnosed in Burkina Faso and Côte d’Ivoire, all of which border Mali (*5**,**14**,**15*). Furthermore, although to date only 1 laboratory-confirmed Lassa fever case has been diagnosed from Mali, there is serologic evidence of a LASV infection in 1971 in a missionary stationed in Mali (*8*). Considering this probable case and the identification of a genetically unique strain of LASV in rodents and a human case from Mali, it is likely that LASV has been present in Mali yet undetected for decades. The nonspecific clinical signs and symptoms of a large proportion of LASV infections, combined with the unfamiliarity of Malian physicians with Lassa fever, suggest that the infection could easily be misdiagnosed. Currently, Lassa fever is not considered in the differential diagnosis for febrile illness in Mali. This report, however, provides evidence of an emerging infectious disease problem with public health effects; thus, Lassa fever diagnostics and surveillance should be implemented at least in the southern regions of Mali.

Soromba and the surrounding area may represent an enzootic hotspot for LASV-infected rodents. However, a greater distribution of LASV in Mali should not be ruled out, particularly in the southern regions of the country where the climate and geography are similar to conditions found in many parts of Guinea and Sierra Leone (Figure 1). Combined with our findings that *M. natalensis* was the predominant species of rodent captured in all 3 villages in Mali suggests that conditions favor LASV circulation. Furthermore, on the basis of the timing of the peak incidence of Lassa fever cases, which typically corresponds with the dry season (*15*), we believe that the proportion of LASV-infected rodents may have been low at the time of these studies and beyond the sensitivity of the limited sampling effort conducted here. Future studies need to address these concerns as well as determine the geographic distribution of LASV-infected rodents in Mali to help focus public health preparedness efforts.
